# The complete chloroplast genome sequence of *Campylandra chinensis* (Liliaceae)

**DOI:** 10.1080/23802359.2018.1491346

**Published:** 2018-07-11

**Authors:** Yafu Zhou, Yuchao Wang, Xinwei Shi, Shaoli Mao

**Affiliations:** Shaanxi Engineering Research Centre for Conservation and Utilization of Botanical Resources, Xi’an Botanical Garden of Shaanxi Province/Institute of Botany of Shaanxi Province, Xi’an, China

**Keywords:** *Campylandra chinensis*, chloroplast genome, medical plant

## Abstract

The complete chloroplast genome of *Campylandra chinensis* from China was analyzed using next-generation sequencing. The chloroplast genome was a 169,419 bp circular molecule and was predicted to contain a large single copy (LSC) of 86,752 bp and a small single copy (SSC) of 21,363 bp, which were separated by a pair of 25,340 bp inverted repeats (IRs). A total of 132 unique genes were annotated, including 86 protein coding genes, 38 tRNA genes, and 8 rRNA genes. Among these genes, 19 genes contained one or two introns. The overall GC contents of the plastid genome was 37.2%. Phylogenetic analysis showed that *C. chinensis* and *Polygonatum* species clustered to one clade with a high bootstrap value at the base of the phylogenetic tree.

*Campylandra chinensis*, a perennial herb which belongs to the genus of *Campylandra* in Liliaceae, is mainly found in moist places of forests or hillsides along streams in south China (Editorial Committee of Flora of China Chinese Academy of Sciences 2000). *C*. *chinensis* is the original plant of the famous medical plant Taibai herbal medicine and is identified by the medical constituents of steroidal compounds, polysaccharides, volatile oils, flavonoids and alkaloids etc, and is extensively used in folk medicine for heat-clearing and detoxifying, and so on (Tang et al. 2016). In this study, the complete chloroplast genome of *C*. *chinensis* was sequenced based on the next-generation sequencing method. The aims of this study were to present the complete chloroplast genome sequences of *C*. *chinensis*, and to provide information for further study and utilization of this important medical species. The annotated chloroplast genome sequence of *C. chinensis* has been deposited into GenBank with the accession number MH356725.

Fresh leaves of *C. chinensis* were collected from Mt. Qinling, Shaanxi, China (33°44′40.01″N, 107°49′15.85″E). The voucher specimens of *C. chinensis* were deposited in Institute of Botany of Shaanxi Province. The complete chloroplast genome DNA was sequenced by Illumina HiSeq 2500 platform (Illumina, CA, USA). ALL of 10,913,983 clean reads were assembled using Velvet 1.2.10 (Zerbino and Birney 2008). The assembled cp genome was annotated using the online program Dual OrganellarGenoMe Annotator (DOGMA; Wyman et al. 2004), combined with manual alterations for the doubtful start and stop codons based on comparison with homologous genes from other sequenced chloroplast genomes in Geneious v 10.1.2 (Biomatters Ltd., Auckland, New Zealand).

The size of chloroplast genome sequence of *Campylandra chinensis* was 158,795 bp in length, including a pair of inverted repeat (IR) regions of 25,340 bp, a large single copy (LSC) region of 86,752 bp, and a small single copy (SSC) region of 21,363 bp. The plastid genome contained a total of 132 unique genes constituting 86 protein coding genes, 38 transfer RNA (tRNA) genes, and 8 ribosomal RNA (rRNA) genes. In addition, there were 19 genes (7 protein coding genes, 8 tRNA genes, and 4 rRNA genes) duplicated in the IR region. Among the 132 unique genes, 12 protein coding genes (*rps16*, *atpF*, *rpoC1*, *ycf3*, *rps12*, *clpP*, *petB*, *petD*, *rpl16*, *rpl2*, *ndhA*, and *ndhB*) and six transfer RNA (*trnG-UCC*, *trnK-UUU*, *trnV-UAC*, *trnL-UAA*, *trnI-GAU*, and *trnA-UGC*) contained one or two introns. The overall GC content was 37.2%, and in the IR regions, LSC and SSC were 43.2%, 35.2%, and 31.2%, respectively. The total length of all 86 PCGs was 79,716 bp, and the overall GC content was 38.1%. The termination codons of all PCGs were typical with TAG, TAA or TGA. The three most-used amino acids in *C. chinensis* were Leu (10.2%), Ile (8.6%) and Ser (7.8%).

Phylogenetic analysis was performed using RAxML (Stamatakis 2014) based on protein coding genes in Liliaceae revealed that *C. chinensis* and *Polygonatum* species clustered to one clade with a high bootstrap value (BP = 96) at the base of the phylogenetic tree (Figure 1).

**Figure 1.**
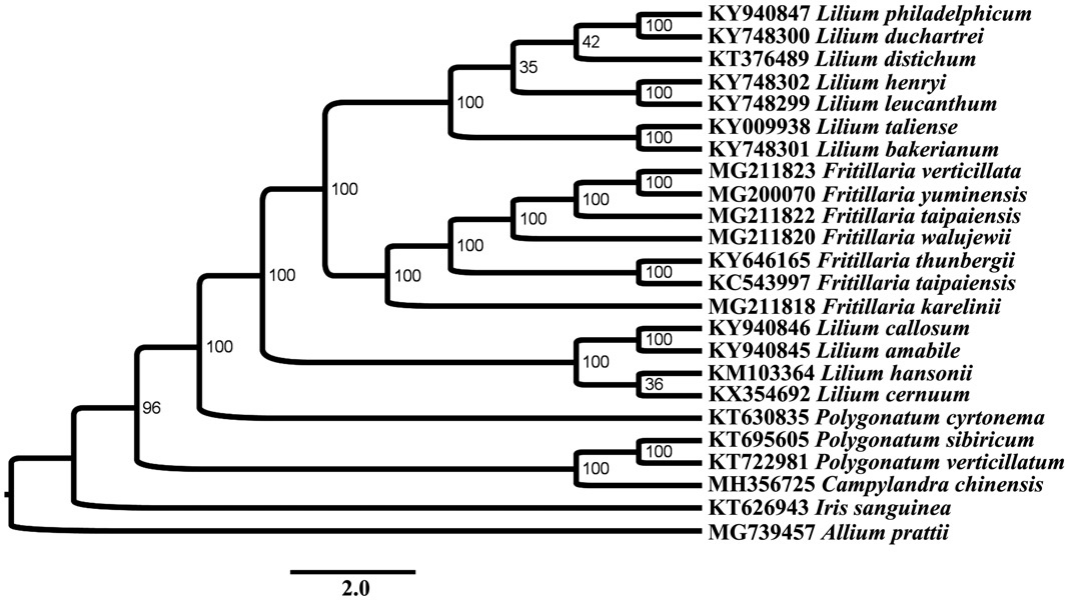
Phylogenetic relationships of Liliaceae using PCGs concatenated dataset.
